# Barriers for Adherence to Diabetic Retinopathy Screening among Saudi Adults

**DOI:** 10.7759/cureus.6454

**Published:** 2019-12-23

**Authors:** Manal Alwazae, Fadwa Al Adel, Atheer Alhumud, Atheer Almutairi, Alhanouf Alhumidan, Hala Elmorshedy

**Affiliations:** 1 Ophthalmology, College of Medicine, Princess Nourah Bint Abdulrahman University (PNU), Riyadh, SAU; 2 Surgery, Ophthalmology, College of Medicine, Princess Nourah Bint Abdulrahman University (PNU), Riyadh, SAU; 3 Medicine, Princess Nourah Bint Abdulrahman University (PNU), Riyadh, SAU

**Keywords:** diabetic retinopathy screening, adherence, barriers, awareness

## Abstract

Background

Diabetic retinopathy (DR) is one of the major complications of diabetes mellitus (DM) and the leading cause of blindness among adults. However, adherence to diabetic retinopathy screening (DRS) significantly reduces blindness. A substantial proportion of diabetics have suboptimal compliance to DRS, which inversely affects their outcomes. Therefore, the aim of this study is to determine the level of adherence to DRS and to explore the factors possibly associated with poor adherence to regular screening among diabetics in Riyadh, Saudi Arabia.

Method

A cross-sectional study was conducted that encompassed 404 adult diabetic patients attending outpatient clinics in four hospitals in Riyadh. A validated, self-administered questionnaire was used for data collection that included five main sections: sociodemographic data, diabetic profile, assessment of knowledge about DR, attitude toward DRS, and barriers to DRS. Data were analyzed by SPSS, version 23 (IBM Corp., Armonk, NY); qualitative variables were described as percentages, and quantitative variables were described as means ± standard deviation (SD). We used the chi-square test to measure the associations between qualitative variables and binary logistic regression analysis to predict the independent barriers to DRS.

Result

The average age of the participants was 54 years, and 69.1% were females. The average duration of diabetes was 12.3 years. Type 2 DM was the most prevalent form of DM (63.6%). DR was reported by 20% of participants. Poor knowledge about DRS was prevalent in 51%. More than one-fifth were never screened for DR. About one-third of participants agreed that cost was an important contributing barrier. Adequate knowledge, increased duration of diabetes, and presence of neurological complications increased independent adherence to screening.

Conclusion

One-fifth of participants reported having DR. Half the participants had poor knowledge about DR, which formed a major barrier against regular screening. However, most participants had positive attitudes about DR screening. Therefore, intervention strategies to increase patients’ awareness of DR might be the cornerstone of ensuring proper adherence to DRS.

## Introduction

Diabetes mellitus (DM) is a chronic debilitating disease that constitutes a substantial problem in both developing and developed nations. This disease is one of the major challenges facing health-care systems due to the remarkable financial burden associated with its multiple comorbidities and high mortality rates [1]. The epidemic of diabetes is a worldwide problem, but its increasing burden is higher in developing countries [2]. According to the International Diabetes Federation in 2017, the estimated number of people (18-99 years old) living with diabetes amounted to 451 million, and it has been projected that the number of diabetics will increase to 693 million by 2045 [3]. The majority of these numerical increments will occur in developing countries [2]. In Saudi Arabia, about one-fourth of people live with diabetes [1].

If uncontrolled, DM leads to many complications in multiple organs, including the eyes. Diabetic retinopathy (DR) is one of the major DM complications and the leading cause of blindness among people 20-74 years old in the United States [4]. Globally, 34.6% of all diabetic patients have some degree of DR, while the annual incidence of DR ranges from 2.2 to 12.7% [5]. Similarly, in Saudi Arabia, the reported prevalence of DR ranges from 31% in the central region to 36% in the western region [6-9].

Multiple risk factors contribute to the development of DR and eventually impaired vision: the type and duration of diabetes, poor glycemic control, and the presence of other comorbidities, such as hypertension and dyslipidemia. Tight glycemic control decreases the onset of DR by 76% and delays the progression of DR by 54% [10]. Frequent screening for the early detection of DR along with appropriate, timely, effective treatment reduces blindness by as much as 98% [11]. In addition, several studies have reported that the control of hypertension and dyslipidemia improved patient outcomes and reduced the risk of blindness by 24% [12].

Helpfully, the global initiative “Vision 2020 The Right to Sight” aims to eliminate avoidable blindness by 2020 [13]. This goal can be achieved by multi-level public health strategies, starting with increasing public awareness and implementing evidence-based national screening programs for all patients with diabetes that ensure timely referral and timely, appropriate treatment. However, these need to be adjusted according to each country’s resource settings and population characteristics [11].

Several studies have reported that a high proportion of patients do not comply with diabetic retinopathy screening (DRS) because of poor knowledge [12,14]. In fact, patients were either unaware or had suboptimal knowledge about DR [15]. For instance, patients confused retinopathy screening and routine eye care, which adversely affected their adherence to the recommended screening [15-19].

Despite the high prevalence of DR in Saudi Arabia, to date, there are insufficient data in the literature regarding adherence to DRS and its association with patients’ levels of knowledge and the potential barriers they face. Therefore, this study determines levels of adherence to DRS and explores the possible contributing factors to poor adherence to regular ocular screening among Saudi adults in Riyadh, Saudi Arabia.

## Materials and methods

A cross-sectional study that encompassed 404 adults of both genders was conducted from September to November 2018. We included diabetic patients with either type of diabetes who were attending outpatient clinics in four governmental hospitals that represent the four different regions of Riyadh: Prince Sultan Military Medical City, King Fahad Medical City, Prince Mohammed Bin Abdulaziz Hospital, and King Abdullah Bin Abdulaziz University Hospital. We calculated the sample size based on the expected level of awareness and adherence to DRS using the free G*Power online software (release 3.1.9.2). Considering a proportion of good knowledge and adherence to DRS as 50% ± 5% and a 95% confidence interval (CI) at a level of significance (α = 0.05) and a power of 80% (β = 0.2), the estimated minimal sample size was 383, which was increased to 404 to compensate for incomplete data. Institutional Review Board (IRB) approval was obtained from all centers included in the study, and all the patients verbally consented prior to enrollment. IRB approval number 18-0226 was obtained from the IRB Committee of Princess Norah Bint Abdulrahman University.

A self-administered questionnaire was adapted from the study of Dervan et al. [20]. After forward and backward translation, a final version was piloted using 20 adult volunteers to confirm its face validity and necessary changes have been made accordingly. Reliability for questions testing attitude (3Q) and knowledge (4Q) were 0.6 and 0.7 respectively using Cronbach’s α test. The questionnaire comprised five main sections: sociodemographic data, diabetic profile, assessment of knowledge, attitude toward DRS, and barriers to DRS. Sociodemographic data included age, gender, marital status, education and income. Income was assessed qualitatively based on four answers (enough and save, enough, not enough and in debt) then grouped into two categories.

Assessment of knowledge was measured by the following questions: Is retinopathy one of diabetes’ complications? Is DR asymptomatic? Are there treatments available for DR? Could regular eye examination prevent the progression of DR? The responses were formatted as “yes,” “no,” and “I do not know.”

The total knowledge score was computed, and the variable was then transformed into a dichotomous measure. Because this is a public survey we considered that adequate knowledge representing those who answered more than two questions (≥70%) and inadequate knowledge representing those who answered only one or two questions (≤50%).

The section measuring attitude toward DRS included three questions that focused on susceptibility to DR, seriousness of the disease, and the benefits of screening. The responses to the attitude questions were on a 3-point Likert scale ranging from 1 (agree) to 3 (disagree). Barriers to DRS were determined by five questions measured on a 3-point Likert scale ranging from 1 (agree) to 3 (disagree) that included the following: cost, family support, fear of results, lack of knowledge about the screening, and screening efficacy.

The statistical analysis was conducted using Statistical Package for Social Science (SPSS) version 23 (IBM Corp., Armonk, NY). The quantitative variables were described in means and SDs, and the qualitative variables were described in proportions. The associations of qualitative variables were assessed by the chi-square test, and P values less than 0.05 were considered statistically significant. A comparison between attendees and non-attendees to DRS was done using the questions related to knowledge, attitude, and barriers. We applied a logistic regression model (forward LR) to determine the independent associations between the possible independent variables and compliance to DRS. We included the following independent variables in the model: knowledge about DR, age, gender, education, income as a measure of socioeconomic status, diabetic complications other than eye complications (cardiac, nephrological, neurological, and diabetic foot), cost, and family support. Odds ratio (OR) and 95% CI were computed.

## Results

Table 1 demonstrates the sociodemographic characteristics of the participants. Participants’ ages ranged from 30 to 60 years with a mean age of 54 ± 15 years. Females accounted for 69.1% of the total sample. The majority of the participants (60.1%) completed a below-high school education, and only 7.9% had low income.

**Table 1:** Sociodemographic characteristics of participants, n = 404

Variables	n (%)
Mean age ± SD	54 ± 15
Females	279 (69.1)
Males	125 (30.9)
Marital status	
Single	133 (32.9)
Married	271 (67.1)
Educational level	
Below high school	243 (60.1)
High school	91 (22.5)
Bachelor and post-graduation	70 (17.4)
Economic level	
Enough and save / enough	372 (92.1)
Not enough / in debt	32 (7.9)

The majority of patients (63.6%) had type 2 DM. 25.7% did not know which type of DM they had. 38.1% controlled their disease by oral hypoglycemic medications, 34.4% were on insulin alone, 23.8% were on both insulin and oral hypoglycemic medications, and 3.7% controlled by diet only. Regarding the non-ocular complications of DM, cardiac complications ranked the highest (27.7%), followed by neuropathy (16.1%), and foot ulcer (11.4%); the least-reported complication was nephropathy (9.2%). One or more diabetic ocular complications were self-reported (46.8%), including cataracts in 31.2%, DR in 20%, glaucoma in 6.2%, and only 2.5% in macular edema. Of note, 53.2% claimed having no diabetes-related ocular complications. More than one-third of participants (36.7%) did not comply with the guidelines to DRS (Table 2).

**Table 2:** Diabetic profile of participants, n = 404

Variables	n (%)	
DM type		
Type 1		43 (10.6)
Type 2		257 (63.6)
I do not know		104 (25.7)
DM treatments		
Insulin		139 (34.4)
Pills		154 (38.1)
Both insulin and pills		96 (23.8)
Diet		15 (3.7)
Mean duration of DM		12.3 (±8)
DM complications		
CVS diseases		112 (27.7)
Neuropathy		65 (16.1)
Nephropathy		37 (9.2)
Diabetic foot / ulcer		46 (11.4)
Ocular complications		189 (46.8)
Diabetic retinopathy		81 (20.0)
Cataract		126 (31.2)
Macular edema		10 (2.5)
Glaucoma		25 (6.2)
Frequency of screening		
Every 3 months		51 (12.6)
Every 6 months		92 (22.8)
Every 12 months		113 (28)
More than every 12 months		60 (14.9)
Never went for screening		88 (21.8)

Table 3 compares knowledge of DR by attendance and non-attendance for screening. Overall, 73.1% of the attendees had adequate knowledge versus 26.9% in non-attendees, P < 0.001. The significant difference in knowledge between attendees and non-attendees held for the individual knowledge questions. Only 39.3% of attendees considered DR an asymptomatic disease versus 26.8% of non-attendees. 61.8% of attendees were aware that DR could be treated, but only 39.5% of non-attendees responded correctly. More than 75% of attendees were aware that DR was a complication of diabetes and that screening helped prevent the progression of the disease versus nearly 50% of non-attendees.

**Table 3:** Awareness of diabetic retinopathy by attendance and non-attendance for screening *P-value by X^2^ test for comparison between attendees and non-attendees to DRS.

Total n = 404			Attendance n = 247 (%)	Non-attendance n = 157 (%)	*P-value
Is retinopathy one of the diabetic complications?					
Yes			(75.7)	(50.3)	<0.001
No			(4.9)	(5.8)	
I do not know			(19.4)	(43.9)	
Could diabetic retinopathy be asymptomatic disease?					
Yes			(39.3)	(26.8)	<0.001
No			(19.8)	(12.1)	
I do not know			(40.9)	(61.1)	
Are there available treatments for diabetic retinopathy?					
Yes			(61.8)	(39.5)	<0.001
No			(7.7)	(1.9)	
I do not know			(30.5)	(58.6)	
Could regular eye examination prevent the progression of diabetic retinopathy?					
Yes			(83.0)	(55.4)	<0.001
No			(4.9)	(5.1)	
I do not know			(12.1)	(39.5)	
Overall level of good knowledge			(73.1)	(26.9)	<0.001
Source of information (n = 404) n (%)					
Physician	288 (71.0)				
Family and friends	145 (35.9)				
Others (Internet, newspaper)	151 (37.4)				

Table 4 illustrates the associations between attitudes toward DRS and the benefits of screening as well as the barriers to complying with DRS. Most of the attendees had good attitudes toward DRS: About 66.8% were worried of going blind from DR, 96% agreed to comply with screening if requested, and 97% valued the importance of screening. In comparison, the percentages of agreement among non-attendees were 58.6%, 86.6%, and 82.8%, respectively. The differences in attitude between attendees and non-attendees were significant (P < 0.05) in readiness for screening when requested and for evaluating the benefits of screening, while it was insignificant in worrying about blindness.

Regarding barriers to DRS, among attendees, cost, lack of family support, and fear of the results of DRS were considered barriers by 26.7%, 19.8%, and 20.6% of attendees versus 29.9%, 17.8%, and 24.8% of non-attendees, respectively (Table 4). However, the difference was not statistically significant. Lack of knowledge about the screening procedure was considered a barrier to screening by 23.5% of attendees versus 32.5% of non-attendees, the difference being statistically significant (P value < 0.001). Also, there was a significant difference between the two groups regarding the perception of the benefits of screening for DR. Only 8.1% of attendees claimed that screening for DR was useless versus 10.8% of non-attendees (P value = 0.002).

**Table 4:** Attitude toward diabetic retinopathy screening by attendance and non-attendance *P-value by X^2^ test for comparison between attendees and non-attendees for screening.

	Total number	Attendance for screening n = 247 (%)	No attendance for screening n = 157 (%)	*P-value
I am worried that I might lose my vision because of diabetes				
Agree		(66.8)	(58.6)	0.15
Natural		(12.1)	(18.5)	
Disagree		(21.1)	(22.9)	
I think it is important to have regular eye examination				
Agree		(97.6)	(82.8)	<0.001
Natural		(1.6)	(13.4)	
Disagree		(0.8)	(3.8)	
If my doctor recommended eye screening for me, I would do it				
Agree		(96.0)	(86.6)	0.003
Natural		(2.4)	(8.3)	
Disagree		(1.6)	(4.1)	
Barriers to diabetic retinopathy screening by attendance and non-attendance for screening				
Cost				
Agree		(26.7)	(29.9)	0.67
Natural		(15.4)	(27.9)	
Disagree		(57.9)	(47.1)	
Lack of family support				
Agree		(19.8)	(17.8)	0.223
Natural		(16.6)	(23.6)	
Disagree		(63.6)	(58.6)	
Fear of result				
Agree		(20.6)	(24.8)	0.207
Natural		(19.4)	(24.2)	
Disagree		(59.9)	(51.0)	
Having no information about the screening procedure				
Agree		(23.5)	(32.5)	<0.001
Natural		(15.8)	(26.1)	
Disagree		(60.7)	(41.4)	
Believing the screening is not effective				
Agree		(8.1)	(10.8)	0.002
Natural		(11.8)	(23.6)	
Disagree		(80.6)	(65.6)	

The results of the logistic regression analysis revealed that adequate knowledge and presence of neurological complications were independently associated with compliance to DRS: (OR = 2.79, 95% CI = 1.80-4.33) and (OR = 2.14, 95% CI = 1.14-4.01), respectively, meaning that adequate knowledge increased compliance to DRS by 179% while the presence of neurological complications increased compliance by 114%. Of note, the regression analysis demonstrated that an increase in the duration of diabetes by one year increased the compliance rate by 5%. The computed Nagelkerke R Square revealed that 15% of the variability of DRS could be explained by the level of knowledge, the presence of neurological complications, and the duration of diabetes (Table 5).

**Table 5:** Adjusted analysis of factors associated with adherence to diabetic retinopathy screening (n = 404) Binary logistic regression model is adjusted for age, gender, education, income as a measure of socioeconomic status, other diabetic complications (cardiac complications, nephrological complications, and diabetic foot), cost, and family support. Nagelkerke R Square = 15%.

Variable factor	OR	95% CI	P value
Good knowledge	2.79	1.80-4.33	<0.001
Neurological complications	2.14	1.14-4.01	0.02
Duration of diabetes	1.05	1.03-1.08	<0.001

## Discussion

Of all DM complications, ocular complications due to DM were the most commonly reported 46.8%. Most patients (79%) had positive attitudes toward DR screening; nevertheless, only half the participants had adequate knowledge about DR. Attendance to screening was found to be suboptimal (61.4%). Similarly, a Japanese cohort study reported that 69.5% of participants visited ophthalmologists on a regular basis [11]. Another study reported that only 57% had attended a routine ocular examination [21]. The most-reported barrier to DRS in our study was cost, as 28% of our sample viewed cost as a barrier to DRS despite our sample being recruited from governmental hospitals that offer free health care services. The cost, in fact, was found to be mainly due to transportation, as opposed to another, similar study that covered 22 states in the United States that showed a lack of medical insurance as the main barrier to DRS [22]. Transportation, however, was also the main concern regarding the cost in another study conducted in Portland, United States, highlighting the need to spread DRS programs in widespread primary health care clinics that can serve patients in different regions [19].

This study revealed that 27% of the sample viewed lack of knowledge as a barrier to DRS. Patients were not aware that DM could affect their eyes by causing DR. Similarly, a systematic review of 35 studies reported that lack of knowledge was a significant barrier to adherence to DRS [16]. This emphasizes the importance of educating patients about DM ocular complications and the relevance of regular screening for those complications.

Fear of results was a barrier to DRS for 22% of our respondents, which was found to be remarkably higher as opposed to another study that reported a much lower percentage (3.6%) [18]. This indicates the need for patient education about the importance and benefits of early detection of DR and early treatment.

Surprisingly, older patients (>60 years old) had better attendance to DRS. This finding was similar to a study conducted in Birmingham in which older patients were more likely to adhere to screening than younger patients [17]. This difference could be because younger patients have less time to attend DRS due to preoccupation with their jobs. In addition, the duration of diabetes could be a moderating factor.

Several studies have reported that most patients that adhere to screening have had DM for more than 10 years [2, 13-14]. In the present study, the adjusted analysis demonstrated that an increase in the duration of diabetes by one year increased the compliance rate by 5%. This may be due to patients better understanding the nature of the disease and the acquired routine and habit of regular screening with time.

In this study, 49% of participants had adequate levels of knowledge about DR. In comparison, a higher level of knowledge was reported in two similar study conducted in Oman (72%) [4]. This result further emphasizes the importance of increasing awareness about DR in the Saudi population.

In further analysis of the level of knowledge among attendees versus non-attendees, 73.1% of attendees had adequate levels of knowledge as opposed to only 26.9% in non-attendees. Likewise, a study done in Hong Kong showed a higher level of awareness among attendees than non-attendees [23]. Of note, 79% of our respondents had positive attitudes toward regular DRS as opposed to another study done in Taiwan that found a slightly higher positive attitude toward regular DRS (90%) [24]. These results reflect the interest of participants in attending DRS despite lack of knowledge. Hence, we expect that raising awareness will dramatically increase compliance to DRS.

The adjusted analysis done in this study further confirmed that adequate knowledge (OR = 2.79, P < 0.001), neuropathy (OR = 2.14, P = 0.02), and longer duration of DM (OR = 1.05, P < 0.001) are all significantly associated with better DRS attendance. Likewise, previous studies conducted in Turkey and Hong Kong found that patients with adequate knowledge were more likely to have regular eye examinations [15,18]. Knowledge deficits have also been identified in other populations; Chou et al. and Cavan et al. reported that level of knowledge had a directly proportional relationship with adherence to DRS [22,25]. Hence, increasing the awareness regarding the importance of DRS needs to be encouraged to improve patient compliance to DRS.

Finally, our study adds genuine information to the few available studies that address the barriers to DRS in Saudi Arabia. The significance of such studies in public health promotion becomes more evident considering the nature and impact of DR, as it is one of the major causes of preventable blindness among adults in Saudi Arabia [26].

## Conclusions

Adherence to DRS among diabetics in Riyadh, Saudi Arabia was found to be suboptimal (61.4%). The most significant barriers to patient adherence to DRS were poor knowledge, shorter duration of the disease, and cost. Nevertheless, our study revealed positive attitudes toward DRS among most patients (79%) in our sample. Therefore, increasing patient compliance with DRS is highly recommended and can be achieved by behavioral interventions, especially raising awareness.
